# The role and diagnostic progress of exosomes in childhood allergic asthma: A review

**DOI:** 10.1097/MD.0000000000045092

**Published:** 2025-10-10

**Authors:** Baohe Liu, Tingting Zu, Lulu Gu, Shuyin Fu, Fanglong Sun, Fuling Wu

**Affiliations:** a Pediatrics Department, Binzhou Medical University Hospital, Binzhou, China.

**Keywords:** allergic asthma, exosomes, immunity, inflammation, miRNA

## Abstract

Allergic asthma in children is a chronic respiratory disease that is characterized by airway inflammation and hyperresponsiveness. Prolonged chronic inflammatory stimulation contributes to the irreversible remodeling of the airways. Currently, there is a high prevalence in childhood, yet a low rate of diagnosis and effective control. This highlights an urgent need to identify a diagnostic tool with high sensitivity and specificity, as well as an effective therapeutic approach. In comparison with the conventional diagnostic and therapeutic techniques for bronchial asthma, exosome technology is capable of detecting minute alterations in asthma-related lung tissue cells at an earlier stage and monitoring airway inflammation in a timely manner. This has the potential to evolve into a highly accurate diagnostic and therapeutic instrument for allergic asthma in children.

## 1. Introduction

### 1.1. Current status of bronchial asthma treatment

Bronchial asthma is a disease that is influenced by a combination of environmental and genetic factors. It is often characterized clinically by the presence of wheezing, shortness of breath and reversible airway spasm. It is estimated that there are more than 300 million individuals with asthma worldwide.^[1]^ Furthermore, there is a correlation between rising levels of environmental pollution and increasing prevalence of asthma.^[2]^ As the most prevalent chronic respiratory disease in children, although it has a lower prevalence rate than adult asthma, asthma has a significantly higher global incidence rate.^[3]^ Furthermore, the level of clinical control of childhood asthma is typically inadequate. Prolonged chronic inflammatory stimulation has been demonstrated to alter airway structure and contribute to irreversible airway remodeling.^[4]^ This study investigate the incidence and prevalence of childhood asthma in recent years, with a particular focus on age-related subgroups (Table 1). The effectiveness of diagnosing and treating asthma in children is therefore of paramount importance. A review of the epidemiological data on asthma in Chinese children and adolescents indicates that the incidence and prevalence of asthma in children under 5 years of age has increased in parallel with the process of urbanization and associated changes in living conditions in China. Conversely, a study on the regression of asthma in children indicates that children diagnosed with asthma within the first 3 years of life are more likely to experience remission from asthma during the 10-year follow-up period. What’s more, over half of the children who wheeze within 3 years of birth are no longer experiencing asthma symptoms at the age of 6 years after treatment.^[5–7]^

**Table 1:** Incidence (/100,000) and prevalence (/100,000) of asthma in Chinese children and adolescents, 1990 and 2019.

Age groups (yr)	Incidence (95% UI)		Rate of change (%)	Prevalence (95% UI)		Rate of change (%)
	1990	2019		1990	2019	
1–	1687.84 (945.28–2771.91)	1694.73 (953.41–2806.00)	0.41 (−3.56 to 3.59)	3030.70 (1679.70–4971.76)	3085.5 (1675.30–5119.74)	1.81 (−1.93 to 5.01)
5–	767.42 (367.12–1307.86)	742.21 (347.36–1276.33)	−3.29 (−7.38 to 0.60)	4396.72 (2626.72–7178.19)	4343.04 (2564.19–7174.89)	−1.22 (−5.09 to 2.23)
10–	417.96 (170.70–705.44)	408.97 (166.11–690.02)	−2.15 (−6.16 to 1.08)	2980.30 (1835.50–4658.51)	2898.71 (1751.58–4600.41)	−2.74 (−6.71 to 0.48)
15–19	270.37 (151.43–424.17)	266.65 (148.99–420.30)	−1.38 (−5.49 to 2.19)	1973.93 (1198.20–3031.94)	1914.71 (1158.20–2923.52)	−3.00 (−7.08 to 0.84)

Allergic asthma represents a clinical subtype of bronchial asthma, approximately 80% of pediatric asthma cases have identifiable allergens.^[8]^ At present, there is no specific treatment available for this condition. However, symptoms can be relieved by environmental interventions, glucocorticoids, anti-allergic drugs and immunotherapy, which includes the use of recombinant humanized anti-immunoglobulin E (IgE) monoclonal antibodies.^[8–10]^ However, the clinical grading of the condition and determination of the requisite medication dosage are primarily based on the patient’s pulmonary function indices and allergen levels. Given the subjective nature of the diagnosis of asthma in children and the potential for repeated stimulation of airway inflammation to lead to airway remodeling as the disease progresses, there is an urgent need to identify more sensitive and specific markers to assist in the management of allergic asthma. For instance, the expression level of miRNA-21 in serum exosomes was found to be statistically significant in patients with varying degrees of asthma.^[11]^ Exosomes derived from lung tissue and exosomes have been demonstrated to play a pivotal role in the regulation of respiratory diseases. Further in-depth studies are anticipated to facilitate the development of specific diagnostic techniques and targeted therapeutic interventions for asthma, while also contributing to advancements in related medical fields.

### 1.2. Overview of exosomes

Exosomes are nanoscale vesicles that can be employed as targeted carriers for the conveyance of substances, including nucleic acids and proteins.^[12]^ It has been demonstrated that exosomes secreted by various cells in the lungs play a pivotal role in the pathogenesis of allergic asthma.^[13]^ Exosomes have been the subject of extensive investigation as potential signaling molecules for the precise treatment of allergic asthma.^[14]^ This article presents a review of the role of exosomes in the pathogenesis of allergic asthma and the research progress in the diagnosis and prognosis of allergic asthma.

Exosomes represent one of the numerous types of exosomes that exhibit a “teato-like” appearance under electron microscopy. They are formed by the wrapping of the cell membrane, devoid of a cytoskeleton, with a diameter ranging from 30 nm to 150 nm.^[15]^ It has been demonstrated that a number of cell types, including eosinophils, macrophages, mast cells and mesenchymal stem cells, are capable of releasing exosomes. These are present in a range of bodily fluids, including blood, saliva and bronchoalveolar lavage fluid.^[16–18]^ Exosomes have a complex composition, containing a variety of DNA, RNA, and a range of lipids, including membrane lipids, cholesterol and numerous other components. The formation of exosomes typically involves the fusion of small vesicles on the cell membrane, with the process dependent on the intracellular transport-essential Endosomal Sorting Complex. This complex recognizes and traps proteins and lipids on the cell membrane, facilitating the formation of exosomes. The process of encapsulation involves the formation of vesicles that translocate towards the interior of the cell, forming intra-lumenal vesicles (ILVs). These ILVs are subsequently transformed into exosomes through 2 distinct pathways. The first pathway involves fusion with lysosomes, which facilitates the degradation of the cargo. The second pathway involves fusion with the cell membrane, enabling the release of the ILVs.^[19]^ The released exosome is distributed to the target cells via various pathways, including blood circulation and tissue fluids. It acts on the target cell through ligands on the exosome surface, interacting with the target cell receptor or being taken up directly through clathrin-dependent or nondependent endocytosis.^[20]^

Exosomes play a significant role in physiological and pathological processes, particularly in the development of inflammatory diseases,^[21]^ cardiovascular diseases,^[22]^ lung diseases^[23]^ and tumors.^[24]^ They have been the subject of extensive research. Exosomes are implicated in a multitude of biological processes, including immune regulation, inflammatory response, cell proliferation and apoptosis, autophagy, and tumor metastasis. The mechanisms through which they exert their effects are being elucidated.^[25]^ It can be reasonably deduced that exosomes have the potential to be utilized in the prevention, diagnosis and treatment of disease.

## 2. The role of exosomes in childhood allergic asthma

**Figure 1.**
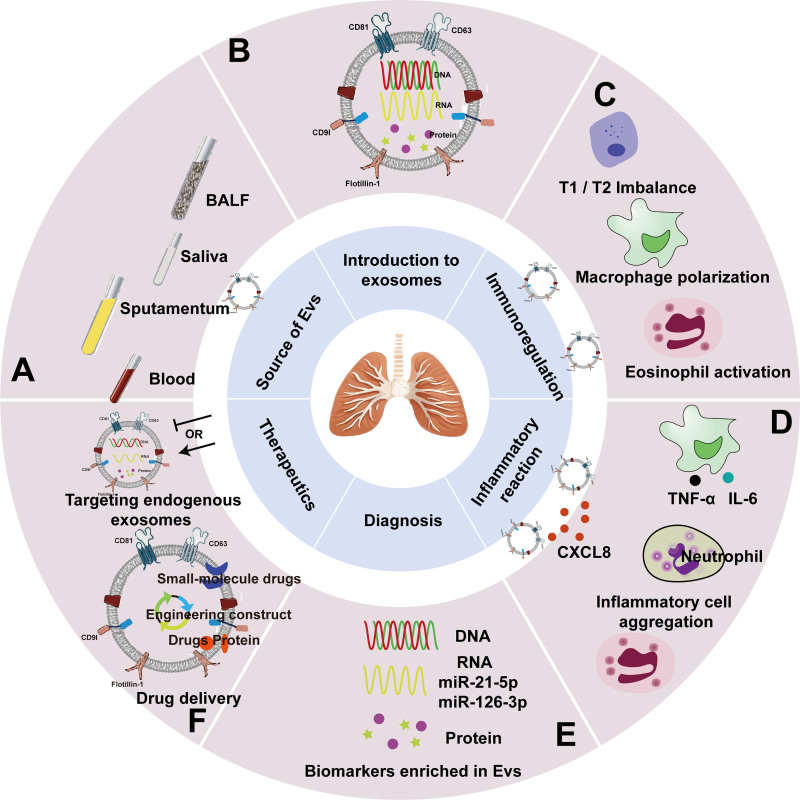
(A) Sources of exosomes: exosomes in blood, sputum, saliva, and bronchoalveolar lavage more intuitively reflect respiratory diseases than systemic exosomes. (B) Introduction of exosomes: express marker proteins such as CD81, carry endogenous proteins and genes. (C) Immunomodulation of exosomes: coordinate the balance of Th1 and Th2 cells, influence macrophage polarization and eosinophil activation. (D) Inflammatory response of exosomes: converging inflammatory cell aggregation, contributing to the secretion of inflammatory factors. (E) Diagnosis of exosomes: exosomes carry cell-specific physiological or pathological proteins, DNA, or RNA molecules. (F) Therapeutic use of exosomes: targeted exosome therapy and exosome-carrying drugs. IL-6 = interleukin-6, TNF-α = tumor necrosis factor-α.

Exosomes typically contain biologically active molecules secreted by primitive cells and are transported to target cells via cellular receptors or cytosolisation. Once in the target cell, the exosomes become communication mediators in the inflammatory cellular microenvironment of the lung.^[26]^ Exosomes are involved in the pathogenesis of allergic asthma, participating in processes such as immune regulation, inflammatory response, smooth muscle contraction and airway remodeling in the lungs^[27]^ (Fig. 1).

### 2.1. Exosome-mediated recognition and processing of allergens

Allergens (e.g., pollen, dust mites, animal dander, etc) directly contribute to the occurrence of asthma attacks or exacerbations in allergic asthma by triggering an abnormal response of the immune system, promoting the release of inflammatory mediators, and airway spasm. Exosomes have the capacity to transport a range of biological molecules, including allergens, major histocompatibility complex molecules, and IgE receptors. These molecules play a role in antigen presentation and immunomodulation. Additionally, exosomes can form synapses with various immune cells, such as dendritic cells, T-cells, B-cells, mast cells, eosinophils, and others. This interaction facilitates the activation of allergen-specific T-cells and the production of IgE antibodies.^[28]^

### 2.2. Exosome-mediated immune imbalance in allergic asthma

Immune cells play a role in regulating allergic responses and the development of airway inflammation. When allergens are exposed to the airways, dendritic cells can capture allergens in the airways and present them to Th2 cells, which promote the proliferation and activation of eosinophils by releasing inflammatory mediators, such as interleukin (IL)-5. This leads to airway inflammation and smooth muscle contraction. However, exosomes are capable of carrying bioactive molecules, including microRNAs and proteins, which interact with immune cells to regulate their function.^[29]^

In allergic asthma, a variety of cells, including type II innate lymphoid cells, are involved in the Th2-induced immune response, which results in inflammatory processes such as airway hyperresponsiveness, mucus secretion, and eosinophil infiltration through the secretion of cytokines IL-4, IL-5, and IL-13.^[30]^ Exosomes have the potential to influence the progression of asthma by regulating the polarization and proliferation of CD4+ T cells via various signaling pathways. For example, exosomes have been demonstrated to inhibit Th2 cell differentiation by regulating the Th1/Th2 balance, thereby attenuating airway inflammatory responses.^[31,32]^ Exosomes derived from mesenchymal stem cells have been demonstrated to inhibit the levels of type II innate lymphoid cells and Th2 cytokines, thereby reducing inflammatory cell infiltration and lung mucus production, and attenuating airway hyperresponsiveness.^[33]^

Additionally, exosomes have been demonstrated to modulate the functionality of other immune cells. It has been demonstrated that macrophages are the most prevalent immune cells in the lung tissue of patients diagnosed with allergic asthma.^[34]^ Following exposure to allergens, the body is capable of producing a range of cytokines, which can induce macrophage differentiation into either classical activated macrophages (M1-type, pro-inflammatory) or alternatively activated macrophages (M2-type, anti-inflammatory).^[35,36]^ It has been demonstrated that exosomes secreted by mesenchymal stem cells facilitate the polarization of macrophages to the M2 phenotype,^[37]^ thereby enabling them to exert anti-inflammatory and immunomodulatory effects in inflammatory lung diseases. Additionally, exosomes derived from polymorphonuclear neutrophils have been demonstrated to regulate immune homeostasis by activating nuclear factor kappa B signaling, which induces M1 macrophage polarization and triggers macrophage colony death.^[38]^

Exosome releases cytokines and chemokines such as IL-10 and tumor necrosis factor-α (TNF-α) that bind to receptors on the surface of eosinophils and mast cells, eliciting intracellular signaling and regulating their activation and release responses.^[29]^ Activated eosinophils promote an inflammatory response through direct release of reactive oxygen species (oxygen), destroying the airway epithelium, while secreting transforming growth factor-β1 to promote smooth muscle proliferation and collagen deposition, and affecting epithelial-to-mesenchymal transition, leading to airway remodeling and fibrosis.^[39]^ Activated mast cells secrete inflammatory mediators histamine, prostaglandin D2, and leukotriene C4, which induce bronchial mucus secretion and mucosal edema by affecting antigen presentation, T-cell differentiation, and leading to allergic symptoms, such as tissue swelling and itching.^[40,41]^ Eosinophil-derived exosomes have been found to chemotaxis eosinophil lung recruitment, increase eosinophil reactive oxygen species and NO production, and upregulate eosinophil oxidative stress and inflammatory processes in allergic asthma through specific protein composition and molecular mechanisms.^[42]^ Mast cell-derived exosomes regulate immune activity by producing cytokines and chemokines (e.g., IL-5, IL-6, IL-10, IL-13, and TNF-α) that influence the differentiation and biological function of adaptive immune cells.^[43]^

### 2.3. Exosomes mediate the inflammatory response in allergic asthma

One of the defining characteristics of allergic asthma is airway inflammation, which results in a significant infiltration of eosinophils, lymphocytes, and other immune cells into the peri-airway tissues. This infiltration mediates the release of various inflammatory mediators, including This results in the release of histamine, interleukins, and lipid intermediates, which cause airway mucosal swelling, increased mucus secretion, and spasm of the airway smooth muscles, leading to airway spasms and respiratory distress.^[8]^ During inflammatory responses, exosomes act as mediators of information transmission between different cells. For example, exosomes induce macrophage activation by mediating the delivery of miR-155 in serum to macrophages, which produce TNF-α and IL-6, thereby exacerbating the inflammatory response in the lungs.^[44]^

Exosomes have the capacity to directly release chemokines, including CXCL8 and CCL2, which serve to guide inflammatory cells to migrate and aggregate at the site of inflammation.^[45–47]^ To illustrate, CXCL8 is capable of attracting neutrophils, eosinophils and other inflammatory cells to migrate towards the site of inflammation, thereby facilitating the inflammatory response. In addition to releasing pro-inflammatory chemokines, exosomes also carry noncoding RNAs that regulate cellular inflammation, thereby exerting anti-inflammatory effects.^[48]^ It has been demonstrated that bone marrow mesenchymal stem cell-derived exosomes, which carry a specific microRNA (miR-127-5p), play a role in septic lung injury by binding to CD64 and inhibiting the neutrophils extracellular trapping networks of neutrophils and the release of inflammatory factors.^[49]^ Furthermore, exosomes are capable of modulating inflammatory responses through the release of proteins, including oxidative enzymes. For example, the exosome protein human leukocyte antigen-G can interact directly with immune cells, affecting the function of natural killer cells and dendritic cells, and inducing apoptosis of activated CD8+ T cells. Furthermore, it participates in the modulation of the balance between Th1 and Th2 cytokines, exerting anti-inflammatory effects.^[50,51]^

### 2.4. Exosomes and airway remodeling

A substantial body of evidence from numerous studies has demonstrated that exosomes play a pivotal role in promoting the repair and regeneration of diverse tissues, thereby offering immense promise for advancing regenerative medicine.^[52]^ Exosomes facilitate cell proliferation and migration of damaged tissues and the proliferation and neogenesis of vascular endothelial cells by transporting growth factors and cell adhesion molecules, enhancing tissue blood supply and accelerating tissue repair.^[53–55]^ Some studies have demonstrated that exosomes can inhibit the Wnt/β-catenin signaling pathway, reduce collagen deposition and reduce scar tissue formation.^[56]^ Furthermore, microRNAs (miRNAs) carried by exosomes (e.g., the microRNAs miR-21, miR-23a, miR-125b, and miR-145) have been demonstrated to influence collagen fiber content and ratio by inhibiting the activation of the transforming growth factor-β pathway, reducing the expression of α-smooth muscle actin, and impeding the differentiation of myofibroblasts.^[57]^ Dong et al^[58]^ demonstrated that hypoxic mesenchymal stem cell-derived exosomes mitigated airway remodeling in a murine model of chronic allergic asthma. This was achieved by comparing lung parenchyma and fiber content measurements in different treatment groups of asthmatic mice.

## 3. Potential clinical use of exosomes in childhood allergic asthma

### 3.1. Exosomes can be used as biomarkers in the diagnosis of allergic asthma in children

The presence of asthma in preschool children has the potential to result in irreversible damage to lung function, which in turn may lead to an increased incidence of chronic lung disease in adulthood. However, the development of various systems in school-age children is not yet mature, the symptoms of wheezing are not yet specific, there is a lack of specific diagnostic methods and indicators, and there is an overdiagnosis of asthma in clinical practice. This is evidenced by the occurrence of abnormal pulmonary function tests during infection, coughing for more than 2 weeks, and the first wheezing attack being diagnosed as asthma. Consequently, an early diagnosis allows for prompt intervention and the alleviation of asthma symptoms, thereby improving the prognosis.

The nucleic acids, proteins or lipids carried by exosomes can be used as primitive reflections of the physiological state of cells. There is an evident disease specificity, which can be employed as noninvasive biomarkers to predict the risk of disease occurrence and is beneficial for early intervention and treatment.^[59,60]^ The implementation of rapid exosome isolation techniques offers novel avenues for personalized treatment in precision medicine.^[61]^

In precision medicine for childhood asthma, exosomes play a role in intercellular communication in the lung microenvironment. Extracellular vesicle collected noninvasively can be used as a “liquid biopsy” to provide information about the disease state.^[62]^ Studies have shown that serum levels of exosomes miR-21-5p, miR-126-3p, miR-146a-5p, and miR-215-5p in children can be used as biomarkers for asthma typing and clinical severity.^[63]^ In addition to serum, the expression levels of exosome-associated miRNAs in bronchoalveolar lavage fluid and sputum also correlate with the severity of allergic asthma.^[64,65]^ It has been proposed that cell-free salivary exosomes can be used as potential biomarkers of asthma, opening up new avenues of research for future studies.^[66]^ In addition, exosomes are not only used for disease diagnosis, but also for monitoring disease progression and evaluating the therapeutic effects of allergic asthma in children in order to further adjust and optimize treatment.^[67]^ This study assesses the relative merits and shortcomings of the most commonly used diagnostic indicators for bronchial asthma in recent years^[68,69]^ (Table 2). Furthermore, it examines the role of exosomes from different cellular sources^[13,70–76]^ in lung tissue in diagnosis and therapy (Table 3).

**Table 2:** Comparison of the advantages and disadvantages of common diagnostic indicators of bronchial asthma.^[67,68]^

Detection indicators	Advantages	Disadvantages
Lung function tests	Objective reflection of the degree of airway patency and lung ventilatory function	1. Lack of unified technical guidelines and reference values for the normal population. 2. Difficulty for younger children to understand and co-operate with the requirements of lung function tests, increasing the time and difficulty of the tests
Exhaled nitric oxide (FeNO) measurement	Rapid, effective and noninvasive method for the detection of biomarkers with high sensitivity and specificity	There are many factors influencing FeNO measurements, with some overlap between asthmatic and non-asthmatic children.
Allergen testing	Serum allergen-specific IgE test, which reflects the severity of the allergic state of asthma patients, is an important basis for the diagnosis of allergic asthma.	Allergy status testing does not directly reflect the presence or absence of allergic inflammation in the airways, but only the individual’s allergy status.
Detection of inflammatory mediators in peripheral blood or sputum	Noninvasive test for early detection of inflammatory mediators while reflecting the degree of inflammation in the disease	Various inflammatory mediators have no clear and uniform diagnostic criteria for asthma in preschool children and lack specificity
Chest imaging	Visualisation of morphological and anatomical changes	Unable to determine functional changes, radiation risk
Bronchoscopic examination	The gold standard for detecting airway inflammation	Invasive operation, costly and nonrepeatable
Genetic polymorphism screening	Helps in early diagnosis and risk assessment to provide personalized treatment	Inaccurate and costly test results

**Table 3:** Role of exosome from different cellular sources in lung tissue in diagnosis and therapy.

Cell source	Mechanisms of action	Diagnostic and therapeutic
Mast cell^[69]^	1. Mast cell surface FcεRI binds to antigen-sensitized specific IgE to activate mast cells and create a pro-inflammatory environment. 2. Recruitment of B cells and T cells to the lungs for action. 3. Interaction with airway smooth muscle induces release of pro-inflammatory factors. 4. Exosome-carried FcεRI binds to free IgE, reducing IgE levels and inhibiting mast cell activation.	1. Determine mast cell activation status by exosome detection of mast cell markers IgE immunoglobulin high-affinity receptor (FcεRI) and c-kit (CD117). 2. The exosome secreted by mast cells binds to free IgE via FcεRI and reduces IgE levels to inhibit mast cell activation.
Dendritic cell^[13,70]^	1. DC-derived exosomes are able to act on distant target cells by direct cell-to-cell contact or paracrine secretion, and are characterized by rapid information transfer and signal amplification. 2. In asthma, the release of thymic stromal lymphopoietin from the airway epithelium induces the release of exosomes from DCs and the expression of OX40 ligand (OX40L, also known as CD252), which promotes the differentiation of CD4 + T-cells into Th2 cells.	1. Determine Th2 cell polarization by detecting exosome OX40L, CD86 expression 2. miRNA in exosomes secreted by DC can stimulate monocytes to produce IL-10, attenuate the response of Th2 cells and inhibit airway hyperresponsiveness.
Eosinophil (type of white blood cell)^[71,72]^	1. Release of major basic proteins, eosinophil cationic proteins, and eosinophil peroxidase induces apoptosis in epithelial cells and expression of asthma-related genes (e.g., POSTN), as well as inducing smooth muscle cell proliferation. 2. Eosinophil-derived exosomes increase the inflammatory properties of eosinophils by upregulating NO and reactive oxygen species in recipient cells. Their autocrine effect is capable of increasing eosinophil adhesion and migration.	1. The number of eosinophil-derived exosomes in asthma patients was significantly higher than that in normal controls, and epithelial cell damage was surmised by detecting the level of shear-type X-box binding protein 1 (sXBP-1) in exosomes. 2. Biological therapy targeting eosinophils improves airway remodeling by reducing exosome miR-338-3p production, inhibiting oxidative stress in epithelial cells, and blocking MAPK and TGF-β signaling pathways.
Macrophage^[73–75]^	1.Macrophage-derived exosomes exert anti-inflammatory or pro-inflammatory effects by regulating macrophage polarization, proliferation, migration, and dedifferentiation of endothelial and smooth muscle cells. 2. Carry bioactive molecules that activate target cell signaling pathways to regulate cell proliferation and transformation.	1. The state and extent of epithelial mesenchymal transition can be inferred by detecting the level of miR-21-5p in exosomes. 2. miR-370, carried by M2-type macrophage-derived exosomes, inhibits airway remodeling and alleviates asthma progression by down-regulating fibroblast growth factor-1 expression.

DC = dendritic cells, FcεRI = high-affinity immunoglobulin E receptor, IgE = immunoglobulin E, MAPK = mitogen-activated protein kinase, TGF-β = transforming growth factor-β.

### 3.2. The potential of exosome in the treatment of allergic asthma

Glucocorticoid therapy is still the mainstay of childhood allergic asthma, and although targeted immunotherapy has brought landmark developments in the treatment of allergic asthma, its efficacy has entered a plateau, and the search for new therapeutic strategies is important to improve the management of allergic asthma in view of the pronounced side effects of hormones.

The discovery of exosomes has changed the understanding of the choreography of the immune response, with exosomes being able to act as independent regulatory units, acting as carrier mediators of intercellular communication and modulating the function and behavior of target cells under physiological and pathological conditions.^[77,78]^ Due to the natural lipid bilayer structure and unique physiological properties of exosomes, these nanoscale vesicles can be modified to encapsulate small molecule drugs, proteins, nucleic acids, etc, or achieve drug loading through membrane proteins on their surfaces to mediate targeted delivery, which not only avoids problems such as immune rejection due to xenotransplantation, but also protects the internal drugs and bioactive molecules from various enzymes, acids and bases, and other environmental interference, prolongs the plasma half-life of the drug, increasing the enrichment of the carrier in the target cells or tissues, and achieving precise delivery of information or precision therapy.^[12,79–81]^ This provides new research ideas for the treatment of asthma.

At present, exosome drug delivery is mainly an exogenous drug delivery route: that is, the exosome is first extracted and purified, and then the therapeutic drug is encapsulated in the exosome. In addition, the efficiency of the exosome drug delivery system to the target site is easily affected by the influence of parent cells and receptor cells, so some of the natural exosomes have defects such as poor targeting. In view of this, specific protein or peptide components can be modified on the membrane surface of exosomes to achieve improved targeting.^[82]^ It has been shown that the loading of triptolide into dendritic cell-derived exosomes successfully attenuated local inflammation and injury in mouse models and did not exhibit significant toxicity, effectively avoiding the multi-organ toxicity of triptolide.^[83]^ Tian et al^[84]^ also demonstrated that curcumin-loaded exosome drug therapy was administered to a mouse model of transient middle cerebral artery embolism after treatment with curcumin. In a mouse model of transient middle cerebral artery embolism treated with curcumin-loaded modified exosomes, the modified exosomes could be successfully targeted to the lesion area of cerebral ischemia and effectively inhibited the inflammatory response and apoptosis in the lesion area.

In addition, given the unique diagnostic properties and specificity of exosomes, targeted therapies against exosomes are also of great research interest. Exosomal miRNAs have been shown to inhibit Th2 cell activation and proliferation through immunomodulation, reduce the release of inflammatory cytokines, and attenuate airway inflammation and hyperresponsiveness.^[85]^ B lymphocyte-derived exosomes can be targeted to induce antigen-specific major histocompatibility complex II-restricted T cell responses.^[86]^ miR-1470 in mesenchymal stem cell-derived exosomes promotes Treg cell differentiation, enhances suppressor T cell function, controls the immune response and attenuates the inflammatory response in patients with asthma.^[87]^ kulshreshtha et al^[88]^ inhibited epithelial cell-derived exosomes with GW4869, which was able to reduce the number of proliferating monocytes in the lung and alleviate asthmatic airway obstruction and mucus secretion. In addition, exosomes were able to enhance the phagocytic activity of alveolar macrophages, thereby facilitating pathogen clearance.^[89]^

## 4. Conclusions and challenges

Allergic asthma is one of the most common chronic diseases threatening the health and life of children. The current conventional diagnostic methods for allergic asthma in children have low sensitivity and weak specificity, which can easily lead to the chronicity of allergic asthma disease, increasing the difficulty of treatment and the risk of late stage. Exosomes are nanoscale particles secreted by various types of living cells that are small, stable and biocompatible, i.e., they can participate in intercellular communication and also serve as biological carriers. In recent years, exosomes have attracted much attention in the early diagnosis, targeted therapy and drug delivery of allergic asthma.

Although exosomes are widely available, purification methods are not standardized and yields are low, not yet sufficient for large-scale application. Researchers still need to explore methods for mass production of exosomes and perfect storage conditions to ensure the stability and efficacy of drugs for clinical use of exosomes. In addition, there is a lack of sufficient clinical data on the use of exosomes in the diagnosis and targeted therapy of allergic asthma, and a lack of consensus given the differences between individual cells and the lack of in-depth understanding of intercellular communication. As a drug delivery vehicle, despite its better biocompatibility, the stability, targeting and safety of exosomes as a drug carrier remains an important consideration, and in-depth studies on its routes of administration, indication of targeted modifications, metabolic pathways and potential toxicity are still needed to ensure its safety in clinical applications.

## Author contributions

**Conceptualization:** Baohe Liu.

**Data curation:** Lulu Gu, Shuyin Fu, Fanglong Sun.

**Writing – original draft:** Baohe Liu, Tingting Zu.

**Writing – review & editing:** Fuling Wu.
